# The burden of chronic pain in transgender and gender diverse populations: Evidence from a large US clinical database

**DOI:** 10.1002/ejp.4725

**Published:** 2024-09-20

**Authors:** Tomasz Tabernacki, David Gilbert, Stephen Rhodes, Kyle Scarberry, Rachel Pope, Megan McNamara, Shubham Gupta, Swagata Banik, Kirtishri Mishra

**Affiliations:** ^1^ Case Western Reserve University School of Medicine Cleveland Ohio USA; ^2^ University Hospitals Urology Institute Cleveland Ohio USA; ^3^ Louis Stokes Cleveland VA Medical Center Cleveland Ohio USA; ^4^ Center for Health Disparities Research and Education Baldwin Wallace University Berea Ohio USA; ^5^ MetroHealth Cleveland Medical Center Cleveland Ohio USA

## Abstract

**Background:**

Chronic pain, affecting approximately 20% of the global population, is the leading cause of disability worldwide. Transgender individuals are disproportionately exposed to chronic pain risk factors compared with the cisgender population. This study compares the incidence of chronic pain between transgender and cisgender individuals and examines the impact of gender affirming hormone therapy, anxiety, and depression on chronic pain.

**Methods:**

The study analysed medical records data of 56,470 transgender men and 41,882 transgender women in the TrinetX database. Six cohorts were created: transgender women either receiving oestrogen or no intervention, transgender men receiving testosterone or no intervention and cohorts of cisgender males and females. Unmatched age‐adjusted incidence rates were calculated. Then cohorts were matched on 22 chronic pain‐associated covariates and the rate of new chronic pain diagnoses was compared between those receiving hormone therapy and those without.

**Results:**

We observed significantly higher rates of chronic pain among transgender individuals compared with cisgender counterparts. Transgender men on testosterone therapy and transgender women on oestrogen therapy exhibited an increased likelihood of chronic pain diagnoses compared with those not receiving hormone therapy. Individuals with anxiety and depression were more likely to be diagnosed with chronic pain.

**Conclusion:**

This study demonstrates a significant burden of chronic pain in transgender individuals, with an increased risk among those receiving hormone therapy. Our study, the first to assess chronic pain in a large cohort of transgender patients, provides support for a potential association between hormone therapy and risk of chronic pain diagnosis. Further research is required to understand causal mechanisms and to develop improved screening and management of chronic pain in transgender populations.

**Significance Statement:**

Our study, featuring the largest cohort of Transgender and Gender Diverse (TGD) individuals assembled to date, reveals critical disparities in chronic pain among TGD populations, notably those on hormone therapy, compared with the cisgender population. It highlights the urgent need for specialized screening and treatment for this vulnerable population, and research into hormone therapy's impact on pain. These insights aim to foster more effective, personalized healthcare, enhancing the well‐being and quality of life for the TGD community.

## 1 INTRODUCTION

Chronic pain is a persistent condition affecting approximately 20% of the world's population and is the leading cause of disability nationwide (Nahin, 2015). In 2021, the Center for Disease Control reported that 51.6 million people (20.9%) in the United States experienced chronic pain, referring to any condition in which aberrant pain lasts for three or more months (Rikard et al., 2023). There is growing recognition that chronic pain is not simply a symptom of underlying pathology, but a disease in its own right, reflected in the 2015 International Classification of Diseases (ICD)‐11 classification system's novel categorization of ‘Chronic Primary Pain’, describing pain disorders which cannot be explained as the secondary symptom of another disease (Treede et al., 2019).

While chronic pain may arise from an initial physical insult or pathology, risk factors like age, gender, race, socioeconomic and employment status, and coexisting conditions like obesity, substance abuse, mental health issues, and sleep disorders contribute to its development (Mills et al., 2019). Additionally, sex hormones are believed to impact pain perception and chronic pain development (Aloisi et al., 2007; Fillingim & Edwards, 2001; Wise et al., 2000).

Transgender and gender diverse (TGD) individuals are persons whose gender identity does not correspond with their sex assigned at birth. Some TGD individuals also may identify outside of the gender binary of male or female. Some TGD individuals may seek gender‐affirming hormone therapy (GAHT) which serves as a critical aspect of transgender healthcare and identity affirmation. Transgender women, who were assigned male at birth and identify as female, may receive exogenous estrogens, often in combination with an anti‐androgen agent. Testosterone is the key hormone in the gender‐affirming treatment of transgender men (assigned female at birth) (Aloisi et al., 2007).

The TGD community is exposed to chronic pain risk factors at disproportionate rates compared with the cisgender population (Abd‐Elsayed et al., 2021). These include disparities in the prevalence of mental health and sleep disorders affecting the TGD community, which have been shown to worsen chronic pain (Boersma & Linton, 2006; Jank et al., 2017; Von Korff et al., 1993). These disparities are compounded by the experience of anti‐transgender discrimination, which may lead to increased instances of comorbid mental health disorders (Abd‐Elsayed et al., 2021; Levit et al., 2021). TGD individuals also have higher rates of substance use disorders, which are also factors associated with increased rates of chronic pain (Hughto et al., 2021). Previous literature points towards a relationship between changes in oestrogen and testosterone levels and changes in pain perception and chronic pain, although this relationship is not well‐understood (Athnaiel et al., 2023; Kato et al., 2020; Wise et al., 2000).

This study utilizes the TriNetX research database to assess chronic pain incidence in TGD individuals versus cisgender patients. Additionally, it aims to compare the impact of several variables on the rate of chronic pain diagnosis in TGD individuals, namely, the impact of GAHT, as well as prior mental health diagnoses such as anxiety and depression, while accounting for chronic pain‐associated risk factors. Given TGD individuals' exposure to chronic pain risk factors, we hypothesize they have a greater risk of chronic pain diagnoses compared with cisgender patients. Additionally, based on the findings in cisgender groups that hormone therapy affects pain perception, we expect differences in chronic pain rates between TGD individuals on GAHT and those who are not. Similarly, we expect that past diagnoses of depression and anxiety will be associated with higher rates of new chronic pain diagnoses among TGD individuals, as previous literature has demonstrated in cisgender populations. This research aims to enhance understanding of chronic pain within TGD populations, identifying key healthcare needs of the TGD community and facilitating informed clinical decision‐making.

## 2 MATERIALS AND METHODS

### 2.1 Data source

For this retrospective cohort study, we used data from the ‘Research’ network on the TriNetX platform (TriNetX, Inc., Cambridge, MA, United States), a clinical research platform that collects real‐time medical records, including demographics, diagnoses, procedures, medications, and laboratory values. This network includes data from around 115.8 million patients and 81 healthcare organizations. Sources of patient records were relatively evenly distributed across the United States (30% Northeast, 21% Midwest, 21% South and 27% West). Data were extracted and analysed from the Research Network on the TriNetX platform on November 21, 2023.

Any data displayed on the TriNetX Platform in aggregate form, or any patient‐level data provided in a data set generated by the TriNetX Platform only contains de‐identified data as per the de‐identification standard defined in Section §164.514(a) of the HIPAA Privacy Rule. The University Hospitals, Cleveland OH, IRB determined research using TriNetX, in the way described here, is not Human Subject Research and therefore IRB exempt.

### 2.2 Study population

We systematically searched the databank to identify TGD patients with records in the study period 2003–2023 based on the presence of ICD‐10 codes F64.0‐F64.9, representing gender identity disorders (GID). Although chart review is the gold standard for the identification of TGD patients, validation studies have demonstrated the high specificity of these codes in electronic health records (EHR/EMR) for identifying TGD individuals (Nik‐Ahd et al., 2023; Proctor et al., 2016; Rich et al., 2021). We were yet more cautious, including only individuals with ≥3 instances of these codes in their medical records. Unfortunately, as TriNetX does not record self‐reported gender identity, we are unfortunately unable to differentiate between transgender and nonbinary individuals in this study. The identified TGD cohort was stratified into four subgroups according to the sex assigned at birth, as recorded in the EMR, and the type of hormone therapy received, if any. The groups included transgender women receiving oestrogen hormone therapy, transgender women with no intervention, transgender men on testosterone therapy, and transgender men with no intervention. The assignment to oestrogen or testosterone hormone therapy categories was determined using prescription codes detailed in Appendix S1. We decided that the use of spironolactone would not be an effective inclusion criterion for GAHT for transgender women, because we cannot distinguish between its use as an anti‐androgen versus for the management of hypertension.

In addition, we constructed two cohorts of cisgender males and females. These cohorts were constructed by including individuals from the TriNetX database with at least three documented inpatient or ambulatory medical visits in their medical record. These cohorts excluded individuals with any ICD‐10 codes indicative of GID, and those with a history of hormone therapy use (Appendix S1), and those with prior diagnosis of chronic pain. Individuals with missing data for sex assigned at birth were omitted from the study.

For our comparison of the relative impact of anxiety and depression on chronic pain, we further divided TGD and cisgender cohorts. For the analysis of depression, each cohort was divided into two groups, those with a diagnosis of recurrent Major Depressive Disorder (MDD) (ICD‐10: F33.0–3, F33.9), documented in at least two medical encounters before the index event, and those without. For the analysis of anxiety disorder, cohorts were divided based on the presence or absence of two documented encounters for Generalized Anxiety Disorder (GAD) (ICD‐10: F41.1).

The index event for Kaplan–Meier analysis for the TGD groups is the earliest use of GAHT in the patient's medical record for the hormone therapy groups, or the first GID diagnosis in the hormone‐naïve groups. For the cisgender individuals, the index event is the first visit recorded in the TriNetX medical record. The TriNetX database included records spanning 2003–2023, although 95% of clinical facts span 2013–2023.

### 2.3 Outcomes

The primary outcome was new diagnosis of chronic pain, identified using specific ICD‐10 codes that are strongly indicative of chronic pain conditions (ICD‐10 codes G89.29, G89.4, F45.41, F45.42). Tian et al. (2013) have previously validated these codes for such purposes, finding a specificity of 99.3% and a PPV of 88.9% (Tian et al., 2013). These codes are used to specify the chronicity of an accompanying localized pain code, and exclude postoperative pain, pain related to neoplasms, and acute pain from our analysis.

### 2.4 Statistical analysis

We compared baseline characteristics between groups using the chi‐square test for categorical variables and the Student's *t*‐test for continuous variables. To assess the burden of chronic pain in TGD and cisgender cohorts, we calculated annualized incidence rates for the period 2013–2023, age‐adjusted against the 2000 U.S. Standard Population (Census P25‐1130). 95% confidence intervals were calculated using the Tiwari modification method (Tiwari et al., 2006). Rate ratios and 95% confidence intervals for the comparison of age‐adjusted rates between groups were calculated using the Byar Approximation.

To control for additional confounders in the evaluation of the potential effects of hormone therapy, the hormone therapy and hormone‐naïve groups were then propensity score matched (PSM) on 22 known chronic pain risk factors and outcomes were compared. Kaplan–Meier analysis was performed to estimate the probability of outcomes from the index date to 10 years or the end of each patient's record. Comparisons between the propensity score matched cohorts were made using both a log‐rank test and Cox proportional hazards models, and scaled Schoenfeld residual test for proportionality, using R's Survival package v3.2–3. Statistical analyses were done within TriNetX and using OpenEpi (Dean, 2013). Statistical significance was set at a two‐sided *p*‐value of <0.05.

### 2.5 Propensity score matching

Using 1:1 nearest neighbour propensity score matching (PSM) with a calliper of 0.1 standard deviations of the propensity score, hormone therapy and hormone‐naïve cohorts were matched on 22 chronic pain‐associated covariates including age, race, preexisting mental health conditions, smoking, alcohol use, and BMI, which have been identified as known risk factors for the development of chronic pain (Mills et al., 2019). These covariates were identified using ICD‐10 codes in the medical record during the time period prior to the index event. As transgender patients receiving hormone therapy may be followed more closely in the clinic than those who are not, the number of ambulatory healthcare visits per patient was also incorporated into the PSM model. For the analyses for the impact of GAD and MDD, PSM was performed using the same methodology, except that MDD and GAD were excluded from the matching criteria for their respective analyses. Balance sheets with full listed covariates are given (Appendix S2).

## 3 RESULTS

### 3.1 Baseline characteristics

We identified 56,470 transgender men and 41,882 transgender women. Among the cohort of 56,470 transgender men, 22,627 were using testosterone therapy, whereas 33,843 were not. The transfeminine cohort included 21,315 individuals using estradiol, and 20,567 who were not using hormone therapy. The average time on continuous treatment for individuals on GAHT was 808.379 days (SD 691.858, Range 1–7039) for transgender men and 818.526 days (SD 817.679, Range 1–7253 days) for transgender women.

In comparison with the cisgender cohorts, transgender cohorts were, on average, younger, predominantly Caucasian, and had a lower average BMI. Additionally, they exhibited higher incidences of depression, anxiety, and sleep disorders (Table 1). Among transgender patients, hormone therapy was associated with higher BMI and age and increased rates of tobacco abuse, depression, anxiety, and sleep disorders. However, there was no significant difference in alcohol abuse or dependence rates between the hormone therapy and hormone‐naïve groups. The baseline characteristics, both prior to and following propensity score matching, are comprehensively detailed in Table 1 and Appendix S1.

**TABLE 1:** Baseline descriptive characteristics of cohorts before propensity score matching.

Characteristics	Transgender women		Transgender men		Cisgender cohorts	
	TWHT (*n* = 21,315)	TWNI (*n* = 20,567)	TMHT (*n* = 22,627)	TMNI (*n* = 33,843)	Cis male (*n* = 53,175,707)	Cis female (*n* = 61,112,392)
Age at index, mean (SD), years	27.3 ± 12.2	26.1 ± 13.9	23.4 ± 10.1	23.1 ± 12.6	36.2 ± 24.7	37.6 ± 24.1
Race						
White	**72.3%**	**66.4%**	**72.5%**	**70.0%**	53%	53%
Black or African American	**8.0%**	**9.6%**	**6.6%**	**6.9%**	12%	12%
Asian or Pacific Islander	2.0%	2.2%	2.4%	2.4%	3%	3%
Unknown race	**11.9%**	**15.4%**	**12.5%**	**14.6%**	26%	25%
Ethnicity						
Hispanic or Latino	8.6%	8.4%	8.8%	8.4%	9%	9%
Not Hispanic or Latino	**75.9%**	**70.6%**	**76.0%**	**73.3%**	49%	48%
BMI, mean (SD)	**26.1** ± **6.8**	**25.0** ± **7.4**	**27.1** ± **7.6**	**25.4** ± **7.4**	28.4 ± 6.12	28.4 ± 7.21
Major Depressive Disorder						
Moderate	**3.5%**	**2.0%**	**3.7%**	**5.3%**	**3.1%**	**1.2%**
Severe	**2.6%**	**1.7%**	**2.8%**	**4.0%**	**3.0%**	**0.7%**
Unspecified	**1.6%**	**1.1%**	**2.3%**	**1.6%**	**0.3%**	**0.5%**
Generalized Anxiety Disorder	**7.6%**	**4.6%**	**10.7%**	**7.5%**	**2.5%**	**4.3%**
Alcohol consumption						
Alcohol abuse	1.6%	1.4%	0.9%	0.6%	**2.1%**	**0.8%**
Alcohol dependence	0.8%	1.0%	0.6%	0.4%	**1.3%**	**0.5%**
Nicotine dependence	**6.5%**	**4.4%**	**4.3%**	**2.2%**	**7.8%**	**5.7%**
Sleep disorders	**7.0%**	**5.4%**	**7.7%**	**6.1%**	**3.9%**	**4.6%**

*Note*: Bold indicates significantly different (*p* < 0.05) between HT and NI groups within the same gender identity before matching; in cis groups, bold indicates significant difference between cis male and female.

Abbreviations: HT, hormone therapy; NI, no intervention; SD, standard deviation; TM, trans men; TW, trans women.

### 3.2 Outcomes

Prior to propensity score matching (PSM), we calculated and age‐standardized the incidence rates of chronic pain to the 2000 Standard Population for the study period. Cisgender males exhibited an age‐adjusted incidence rate of 1821 per 100,000 person‐years. In contrast, cisgender females displayed a significantly higher rate of 1883 per 100,000 person‐years (Table 2).

**TABLE 2:** Comparison of age adjusted incidence rates between trans and gender diverse individuals receiving or not receiving gender‐affirming hormone therapy compared with cisgender cohorts.

	Cases	PY, years	Age‐standardized IR per 100,000 PY (95% CI)	Adjusated RR HT vs NI (95% CI)	Adjusted RR compared with cis cohort(95% CI)
TWHT	1882	70,143	3309.92 (3160, 3459)	1.02 (0.97, 1.07)	**1.82 (1.72, 1.92)**
TWNI	1337	48,065	3259.98 (3085, 3435)	1 [Reference]	**1.79 (1.69, 1.90)**
TMHT	2002	58,239	3922.99 (3751, 4095)	**1.26 (1.20, 1.32)**	**2.08 (1.97, 2.20)**
TMNI	1707	60,384	3125.01 (2977, 3273)	1 [Reference]	**1.66 (1.57, 1.76)**
Cis male	1,696,772	89,515,887	1821.31 (1821, 1827)		1 [Reference]
Cis female	2,410,212	125,062,008	1883.46 (1881, 1886)		1 [Reference]

*Note*: Bold indicates *p* < 0.05 between cohort and reference group. Trans groups are compared with cis cohort corresponding to their natal sex.

Abbreviations: CI, confidence interval; HT, hormone therapy; IR, incidence rate; NI, no intervention; PY, person‐years of follow‐up; RR, risk ratio; TM, trans men; TW, trans women.

#### 3.2.1 Analysis 1. Comparison of age‐adjusted incidence of chronic pain between TGD and cisgender cohorts

In our TGD cohorts, we observed notably higher rates of chronic pain diagnoses compared with their cisgender counterparts. Specifically, transgender women receiving GAHT had an incidence rate of 3309 per 100,000 person‐years. This rate was significantly higher than that of cisgender males (Relative Risk [RR] 1.82; 95% Confidence Interval [CI]: 1.72, 1.92). Transgender women naïve to oestrogen therapy showed a similar trend, with an incidence rate of 3260 per 100,000 person‐years, again significantly exceeding that of cisgender males (RR 1.79; 95% CI: 1.69, 1.90). Notably, the incidence of chronic pain between transgender women receiving hormone therapy and those naïve to hormone therapy did not differ significantly prior to PSM (Table 2).

For transgender men on testosterone, the incidence rate was markedly higher at 3923 per 100,000 person‐years when compared with cisgender females (RR 2.08; 95% CI: 1.97, 2.20). Transgender men naïve to hormone therapy also exhibited a higher incidence rate of chronic pain than cisgender females (RR 1.66; 95% CI: 1.57, 1.76). Furthermore, transgender men on hormone therapy had a significantly higher rate of chronic pain compared with those naïve to therapy (RR 1.26; 95% CI 1.20, 1.32) (Table 2).

#### 3.2.2 Analysis 2. Risk of chronic pain between hormone therapy and hormone Naïve cohorts after propensity score matching

**FIGURE 1:**
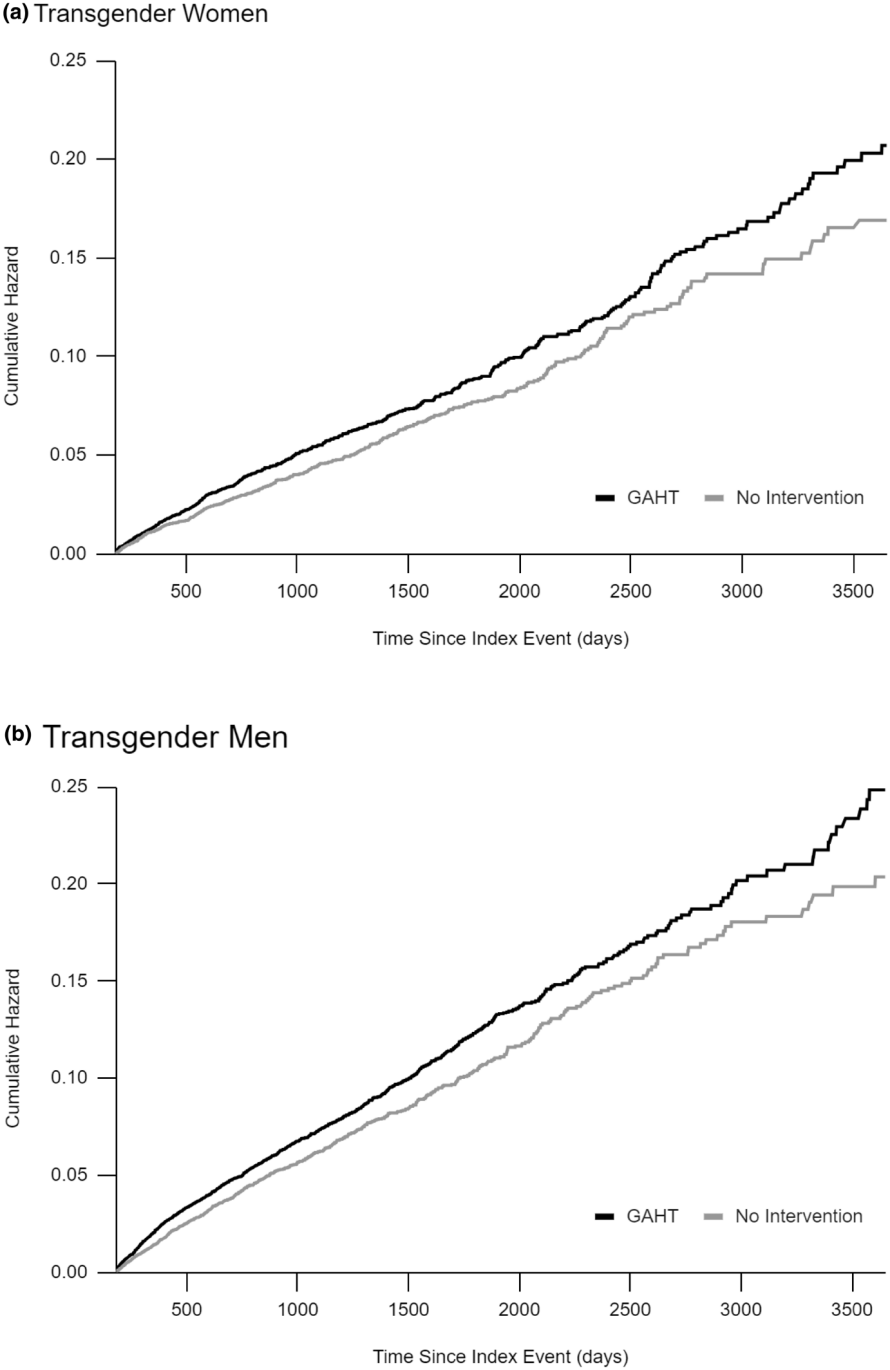
10‐Year Kaplan–Meier Estimate of Cumulative Risk of Chronic Pain Diagnosis. 10‐Year Kaplan–Meier estimate of the cumulative risk of chronic pain diagnosis. (A) Risk is compared between transgender women receiving gender affirming hormone therapy (GAHT) compared with those naïve to hormone therapy (No Intervention). (B) Risk is compared between transgender men receiving gender affirming hormone therapy (GAHT) compared with those naïve to hormone therapy (No Intervention).

Following propensity score matching, the cohort sizes adjusted to 17,352 for both transgender female cohorts, and 22,037 for the transgender men, with standardized mean differences for all covariates below 0.035. Post‐matching analyses revealed that recipients of hormone therapy had an increased likelihood of chronic pain diagnosis. Cox proportional hazards models indicated a 20% increased hazard of new chronic pain diagnoses for transgender men receiving hormone therapy compared with transgender men naïve to hormone therapy (HR: 1.20; 95%CI: 1.096, 1.312). Similarly, transgender women receiving hormone therapy were more likely to receive a new diagnosis of chronic pain when compared with transgender women not using hormone therapy (HR: 1.194; 95% CI: 1.063, 1.331) (Table 3). These data are further illustrated in Figure 1.

**TABLE 3:** Comparative statistical analysis of chronic pain outcomes after propensity score matching.

Group	Cohort statistics			Log rank test		Hazard ratio
	Cohort size	Patients with chronic pain, no. (%)	X^2	*p*	HR	95% CI
TMHT	22,037	1253 (5.686%)	15.674	**<0.0001**	**1.20**	**(1.096, 1.312)**
TMNI	22,037	768 (3.485%)				
TWHT	17,352	791 (4.559%)	9.192	**0.0024**	**1.19**	**(1.063, 1.331)**
TWNI	17,352	496 (2.858%)				

*Note*: Bold indicates statistical significance (*p* < 0.05).

Abbreviations: CI, confidence interval; HR, hazard ratio; HT = hormone therapy; NI, no intervention; TM, trans men; TW, trans women.

#### 3.2.3 Analysis 3. Risk of chronic pain between MDD versus non‐MDD cohorts after propensity score matching

We assessed the impact of Major Depressive Disorder (MDD) on the incidence of chronic pain among transgender and cisgender cohorts, using Cox proportional hazard ratios after propensity score matching (PSM).

Among cisgender men, individuals with MDD had a significantly higher hazard of chronic pain when compared with those without this diagnosis (HR: 1.704, CI: 1.678, 1.73). Similar findings were seen when comparing cisgender women with and without depression (HR: 1.942, CI: 1.921, 1.963) (Table 4).

**TABLE 4:** Cox proportional hazard ratios comparing chronic pain among individuals with and without Major Depressive Disorder.

Group	Cohort statistics		Cox proportional Hazard ratio	
	Cohort size	Patients with chronic pain, no. (%)	HR	95% CI
TW with MDD	3254	179 (5.50%)	**1.451**	**(1.156, 1.822)**
TW without MDD	3254	127 (3.90%)		
TM with MDD	6398	399 (6.24%)	**1.348**	**(1.164, 1.561)**
TM without MDD	6398	324 (5.06%)		
Cis male with MDD	150,894	44,880 (29.74%)	**1.704**	**(1.678, 1.73)**
Cis male without MDD	150,894	25,806 (17.10%)		
Cis female with MDD	264,718	95,992 (36.26%)	**1.942**	**(1.921, 1.963)**
Cis female without MDD	264,718	48,599 (18.36%)		

*Note*: Bold indicates statistically significant difference between group with depression compared with group without depression (*p* < 0.05). MDD = 2 or more visits documenting Major Depressive Disorder prior to the index event.

Abbreviations: TW, transgender woman; TM, transgender man; HR, Hazard Ratio; CI, confidence interval.

Transgender women with major depressive disorder, when compared with those without depression, had a significantly higher hazard of chronic pain (HR: 1.451, CI: 1.156, 1.822) (Table 4). This increased risk was not significantly different from the increased hazard observed in cisgender men.

Among transgender men, individuals with major depressive disorder had a significantly higher hazard of chronic pain when compared with those without depression (HR: 1.348, CI: 1.164, 1.561). Notably, the increased hazard associated with MDD in the transgender male cohort was significantly lower compared with the increased hazard observed in the cisgender female cohort (Table 4).

#### 3.2.4 Analysis 4. Risk of chronic pain between GAD versus non‐GAD cohorts after propensity score matching

A similar stratification was performed, dividing the cisgender and TGD male and female hormone therapy and hormone naïve cohorts by the presence of 2 or more diagnoses of GAD prior to the index event. Among cisgender men, individuals with GAD diagnoses had a significantly higher hazard of chronic pain when compared with those who did not (HR: 1.646, CI: 1.633, 1.659). There was a similarly elevated hazard of chronic pain associated with GAD among cisgender women (HR: 1.59, CI: 1.581, 1.599) (Table 5).

**TABLE 5:** Cox proportional hazard ratios comparing chronic pain among individuals with and without generalized anxiety disorder.

Group	Cohort statistics		Cox proportional hazard ratio	
	Cohort size	Patients with chronic pain, no. (%)	HR	95% CI
TW with GAD	4256	174 (4.09%)	**1.65**	**(1.296, 2.1)**
TW without GAD	4256	107 (2.51%)		
TM with GAD	7308	326 (4.46%)	**1.478**	**(1.249, 1.75)**
TM without GAD	7308	231 (3.16%)		
Cis Male with GAD	808,310	177,506 (21.96%)	**1.646**	**(1.633, 1.659)**
Cis Male without GAD	808,310	99,847 (12.35%)		
Cis Female with GAD	1,824,172	376,612 (20.65%)	**1.59**	**(1.581, 1.599)**
Cis Female without GAD	4,577,207	437,721 (9.56%)		

*Note*: Bold indicates statistically significant difference between group with generalized anxiety disorder compared with group without generalized anxiety disorder (*p* < 0.05). GAD = 2 or more visits documenting Generalized Anxiety Disorder prior to the index event.

Abbreviations: CI, confidence interval; HR, hazard ratio; TM, transgender man; TW, transgender woman.

Among transgender women, individuals with GAD diagnoses had a significantly higher hazard of chronic pain when compared with those who did not (HR: 1.65, CI: 1.296, 2.1). This increased risk was not significantly different from the increased hazard observed in cisgender men (Table 5).

Among transgender men, individuals with GAD diagnoses had a significantly higher hazard of chronic pain when compared with those who did not (HR: 1.478, CI: 1.249, 1.75). This increased risk was not significantly different from the increased hazard observed in cisgender women (Table 5).

## 4 DISCUSSION

Our study examines the burden of chronic pain among Transgender and Gender Diverse (TGD) individuals, particularly focusing on those receiving gender‐affirming hormone therapy. The observed higher incidence of chronic pain among TGD population in our dataset compared with cisgender counterparts are consistent with the findings of Zajacova et al., (2023), who noted similar disparities (Zajacova et al., 2023). These disparities may stem from a range of factors including minority stress, discrimination, and higher prevalence of mental health conditions (Hughto et al., 2021; Levit et al., 2021).

Our study adds important insights on the pattern of chronic pain among TGD people after adjusting for known confounders. We noted a higher likelihood of chronic pain diagnosis in individuals on GAHT, when compared with those naïve to GAHT. Importantly, this pattern was observed in both TGD men receiving testosterone therapy and TGD women undergoing oestrogen therapy. The association between GAHT and increased chronic pain risk observed in our study aligns with existing literature discussing the impact of sex hormones on pain modulation (Aloisi et al., 2007; Fillingim & Edwards, 2001; Wise et al., 2000). However, our findings also highlight the complexity of this relationship, particularly when comparing the effects of testosterone and oestrogen across cisgender and TGD populations.

Our findings align with previous data supporting that oestrogen HRT is associated with increased incidence of chronic pain. When interpreting these findings in the context of transgender health, it should be noted that doses of oestrogen used for feminization are significantly higher than those used in the post‐menopausal population. A survey by Wise et al. found that post‐menopausal women on hormone replacement therapy (HRT) reported higher rates of orofacial pain than those not on HRT (Wise et al., 2000). Similarly, Fillingim et al. observed that cisgender women on HRT exhibited lower experimental pain thresholds and tolerance than those not receiving HRT and cisgender men (Fillingim & Edwards, 2001). Additionally, a 1998 study reported higher incidences of chronic low back pain among women on HRT, when compared with those not receiving this treatment (Brynhildsen et al., 1998). In a study of transgender women, Aloisi et al. reported higher rates of chronic pain in transgender women on oestrogen therapy (Aloisi et al., 2007). These studies suggest an association between the use of oestrogen HRT and changes in chronic pain perception and the development, a pattern that is recapitulated in our findings.

Notably, our results differ from studies suggesting a protective effect of testosterone against chronic pain in cisgender men (Athnaiel et al., 2023; Kato et al., 2020), indicating a unique interaction of hormone therapy and pain perception in TGD individuals. There is some data to support our findings. Notably, a study by Zwickl et al found that 72.2% of the transgender men they surveyed reported increased pelvic pain while receiving testosterone therapy (Zwickl et al., 2023). Other studies have noted that transgender men receiving routine cervical cancer screening report far higher levels of pain during the procedure while on testosterone therapy, which may be due to testosterone's effect on pelvic floor musculature (Dhillon et al., 2020). Furthermore, based on a study in Israel, Levit et al reported that transgender men had a nearly 6‐fold higher prevalence of fibromyalgia compared with cisgender women, despite the use of testosterone therapy (Levit et al., 2021). An interplay of natal hormone exposure and later‐life hormone therapy might explain these variations. Levit et al. posits that in‐utero exposure female hormones may predispose transgender men to chronic pain conditions, despite subsequent hormone replacement therapy (Levit et al., 2021). Our findings underscore the need for further studies to explain the mechanism behind elevated rates of chronic pain after testosterone GAHT in TGD men.

In interpreting our findings, the role of psychosocial factors in chronic pain among Transgender and Gender Diverse (TGD) individuals undergoing hormone therapy is pivotal. We observed that for TGD males and females, prior diagnosis of MDD and GAD were associated with an increased risk of chronic pain diagnosis. The impact of MDD and GAD was generally not significantly different between the TGD and cisgender cohorts, with the exception of MDD among transgender men, which was associated with a lower increase in chronic pain risk compared with cisgender women. This recapitulates prior literature, suggesting that depression and anxiety are strongly associated with chronic pain among cisgender populations (Boersma & Linton, 2006; Von Korff et al., 1993; Woo, 2010).

This insight is of particular importance, as adverse experiences of discrimination and psychological distress may lead to increased rates of depression and anxiety among the TGD population (Abd‐Elsayed et al., 2021; Levit et al., 2021). While gender dysphoria and related psychological distress often improve with the initiation of GAHT, individuals may face increased discrimination and psychosocial stress, especially during the public transition phase (Levit et al., 2021; Verbeek et al., 2020). Such experiences are linked to adverse health outcomes like chronic pain (Craig et al., 2020). Additionally, negative healthcare experiences, ranging from treatment refusal to harassment, reported by 33% of TGD individuals (James et al., 2016), further compound these challenges. Therefore, addressing discrimination and anti‐transgender stigma and its resulting impact on mental health is critical in the effective management and understanding of chronic pain in this population.

### 4.1 Limitations

Several limitations of our study must be acknowledged. First, the estimation of incidence rates from EMR data is complex, and calculated rates may differ depending on the database and calculation methods used (Bagley & Altman, 2016; Rassen et al., 2018). Our study compared our calculated incidence rates with other cohorts on the same platform to eliminate this source of error.

Our propensity score matching may not fully account for unmeasured or residual confounding. Unavailable data points such as socioeconomic and employment status could potentially introduce additional confounding. Our reliance on ICD‐10 codes for chronic pain, although selected for their high specificity, may not capture all chronic pain conditions experienced within our study cohort (Tian et al., 2013). They also do not provide data on the locations or aetiologies of chronic pain conditions. However, we believe that this design choice is justified, as using other codes such as those for ‘Low Back Pain’, do not allow us to confirm chronicity of these pain conditions. Overall, this limitation will likely lead to an underestimate of chronic pain incidence across all study groups and does not take away from our primary finding that the relative burden of chronic pain in the transgender population is significantly higher than the cisgender cohorts.

For our analysis of the impact of MDD and GAD, limitations of our dataset prevented further analysis and stratification of the cohorts by duration or other features of a patient's MDD or GAD, which may lead to heterogeneity among the cohorts. This limitation of the dataset may lead to qualitative differences in individuals experience of these mental health conditions, which may impact the ability to compare hazard rates between TGD and cisgender cohorts. It does not impact the hazard ratios of MDD/GAD versus non‐MDD/non‐GAD cohorts.

Healthcare access disparities among TGD populations may contribute to an underrepresentation of certain covariates and outcomes, although we sought to control for this by including healthcare utilization in our PSM model.

Although TriNetX offers a field denoting sex assigned at birth, an individual's identified gender may have been reported instead. Since sex assigned at birth carries a higher weight biologically when it comes to medical decisions, we believe most EMR systems still register patients with their sex assigned at birth. Lastly, while our identification codes for TGD patients display high specificity, their sensitivity is somewhat lower (Blosnich et al., 2018; Nik‐Ahd et al., 2023; Proctor et al., 2016; Rich et al., 2021). This indicates that it is likely we may not have captured the entirety of the TGD population.

### 4.2 Strengths

Despite these limitations, our study has several key strengths. First, the size of our cohort, larger than any prior investigation, lends weight to our findings in the context of hormone therapy's impact on TGD populations. Our dataset offers a wide geographical and racial diversity, which contributes to a more representative understanding that may be difficult to capture in other studies. Additionally, our methodology provides the capacity to control for an array of potential confounders, enhancing our analysis of hormone therapy, MDD, and GAD, and chronic pain among TGD individuals.

## 5 CONCLUSION

Our research highlights disparities in the cumulative risk and burden of chronic pain in Transgender and Gender Diverse (TGD) individuals compared with their cisgender counterparts. Additionally, it suggests an association between hormone therapy and an increased risk of chronic pain in the TGD population. Lastly, our analysis confirms that MDD and GAD are associated with an increased risk of chronic pain among TGD individuals. These findings emphasize the necessity for comprehensive screening and enhanced treatment strategies for TGD individuals, particularly those undergoing hormone therapy. The study underscores the need for further investigation into the relationship between sex hormones and pain, especially for TGD individuals on gender‐affirming hormone therapy. A deeper understanding of these interactions could pave the way for more personalized and effective healthcare approaches for TGD patients. Overall, this study contributes to the expanding knowledge of chronic pain within the transgender community, aiming to bolster health outcomes and quality of life for TGD individuals.

## AUTHOR CONTRIBUTIONS

**Tomasz Tabernacki**: Conceptualization, Data Curation, Formal Analysis, Methodology, Project Administration, Writing‐original draft, Writing review and editing. **David Gilbert**: Methodology, Writing‐Original Draft, Writing‐Review and Editing. **Stephen Rhodes**: Methodology, Formal Analysis, Data Curation, Validation, Supervision. **Kyle Scarberry**: Writing‐Review and Editing. **Rachel Pope**: Writing‐Review and Editing. **Megan McNamara**: Writing‐Review and Editing. **Shubham Gupta**: Writing‐Review and Editing. **Swagata Banik**: Writing‐Review and Editing, Validation. **Kirtishri Mishra**: Writing‐Review and Editing, Supervision, Project Administration, Validation.

## FUNDING INFORMATION

This publication was made possible through the support of the Clinical Research Center of University Hospitals Cleveland Medical Center (UHCMC) and the Case Western Reserve University Clinical and Translational Science Collaborative (CTSC) 4UL1TR000439.

## CONFLICT OF INTEREST STATEMENT

The authors declare that they have no known competing financial interests or personal relationships that could have appeared to influence the work reported in this paper.

## Supporting information


Appendix S1.

Appendix S2.
