# Supplementary material for: The burden of chronic pain in transgender and gender diverse populations: Evidence from a large US clinical database
Source: Eur J Pain. 2024 Sep 20;29(2):e4725. doi: 10.1002/ejp.4725 (PMC11671315; doi:10.1002/ejp.4725)
Supplement: Supplementary file 1 — Appendix S1. [file EJP-29-0-s002.docx]

**Appendix A- Cohort and Outcome Criteria:**

Cohort Construction- Terms Denoting Gender Identity Disorders

|  | ICD-10-CM Code |
| --- | --- |
| Transsexualism | F64.0 |
| Dual role transvestism | F64.1 |
| Gender identity disorder of childhood | F64.2 |
| Other gender identity disorders | F64.8 |
| Gender identity disorder, unspecified | F64.9 |

Cohort Construction-Terms Denoting Testosterone Gender Affirming Hormone Therapy:

|  | ICD-10-CM/RXNORM/VA Class/HCPCS Code |
| --- | --- |
| ANDROGENS/ANABOLICS | VA:HS100 |
| testosterone | RXNORM:10379 |
| Injection, testosterone cypionate, 1 mg | HCPCS:J1071 |
| Injection, testosterone enanthate, 1 mg | HCPCS:J3121 |
| Injection, testosterone undecanoate, 1 mg | HCPCS:J3145 |

Cohort Construction-Terms Denoting Estrogen Gender Affirming Hormone Therapy

|  | ICD-10-CM/RXNORM/VA Class/HCPCS/ATC Code |
| --- | --- |
| ESTROGENS | ATC:G03C |
| Estrogens | ATC:L02AA |
| ESTROGENS | VA:HS300 |
| estrogens, esterified (USP) | RXNORM:214549 |
| estrogens | RXNORM:4100 |

Outcome Measures-Terms Denoting Chronic Pain

|  | ICD-10-CM Code |
| --- | --- |
| Other chronic pain | ICD10CM:G89.29 |
| Chronic pain syndrome | ICD10CM:G89.4 |
| Chronic psychogenic pain | ICD10CM:F45.4 |
