# Supplementary material for: The burden of chronic pain in transgender and gender diverse populations: Evidence from a large US clinical database
Source: Eur J Pain. 2024 Sep 20;29(2):e4725. doi: 10.1002/ejp.4725 (PMC11671315; doi:10.1002/ejp.4725)
Supplement: Supplementary file 2 — Appendix S2. [file EJP-29-0-s001.docx]

**Appendix B-Cohort Baseline Characteristics and Propensity Score Matching Balance Tables:**

Trans Women On Estrogen Therapy vs Trans Women Naïve to Hormone Therapy

| Cohort 1 and cohort 2 patient count before and after propensity score matching | | | | | | | | | | | |
| --- | --- | --- | --- | --- | --- | --- | --- | --- | --- | --- | --- |
|  | | Cohort | | | Patient count before matching | | | | Patient count after matching | | |
|  | | 1 – Trans Women on Estrogen Hormone Therapy | | | 21,630 | | | | 17,352 | | |
|  | | 2 - Trans Women Naïve to Hormone Therapy | | | 20,648 | | | | 17,352 | | |
| Propensity score density function - Before and after matching (cohort 1 - black, cohort 2 - grey) | | | | | | | | | | | |
|  |  | 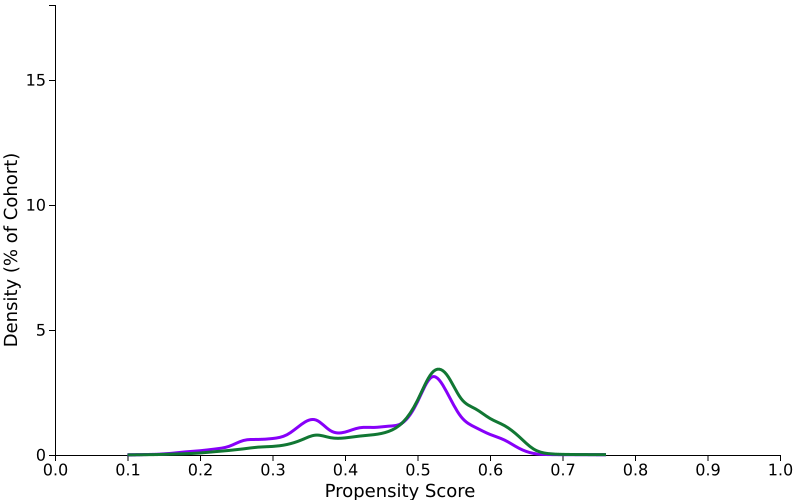 | | | | 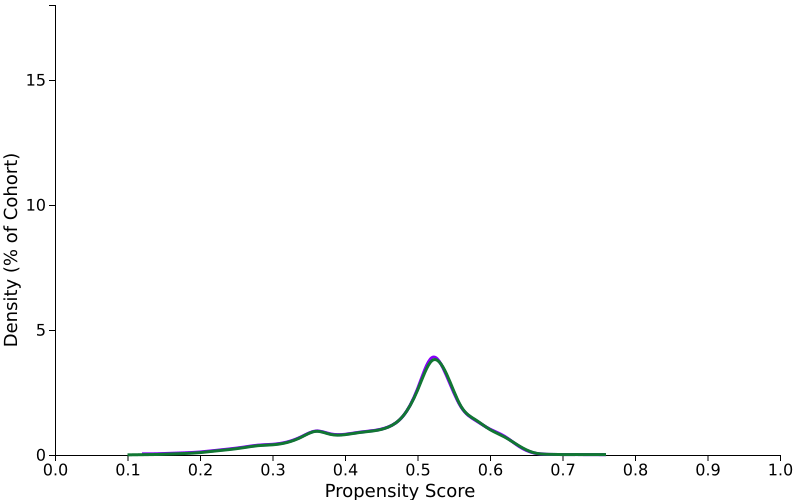 | | | | | |
| Cohort 1 (N = 21,630) and cohort 2 (N = 20,648) characteristics before propensity score matching | | | | | | | | | | | |
|  | Demographics | | | | | | | | | | |
|  |  | Cohort | |  | Mean ± SD | | Patients | % of Cohort | | P-Value | Std diff. |
|  |  | 1 2 | Age | Current Age | 31.1 +/- 12.9 30.1 +/- 14.5 | | 21,531 20,429 | 100% 100% | | <0.001 | 0.070 |
|  |  | 1 2 | AI | Age at Index | 27.3 +/- 12.2 26.1 +/- 13.9 | | 21,531 20,429 | 100% 100% | | <0.001 | 0.088 |
|  |  | 1 2 | 2106-3 | White |  | | 15,507 13,524 | 72.0% 66.2% | | <0.001 | 0.126 |
|  |  | 1 2 | UNK | Unknown Race |  | | 2,536 3,132 | 11.8% 15.3% | | <0.001 | 0.104 |
|  |  | 1 2 | UN | Unknown Ethnicity |  | | 3,519 4,414 | 16.3% 21.6% | | <0.001 | 0.135 |
|  |  | 1 2 | 2186-5 | Not Hispanic or Latino |  | | 16,188 14,307 | 75.2% 70.0% | | <0.001 | 0.116 |
|  |  | 1 2 | 2135-2 | Hispanic or Latino |  | | 1,824 1,708 | 8.5% 8.4% | | 0.683 | 0.004 |
|  |  | 1 2 | 2054-5 | Black or African American |  | | 1,749 1,977 | 8.1% 9.7% | | <0.001 | 0.055 |
|  |  | 1 2 | 2028-9 | Asian |  | | 430 448 | 2.0% 2.2% | | 0.161 | 0.014 |
|  | Diagnosis | | | | | | | | | | |
|  |  | Cohort | |  | Mean ± SD | | Patients | % of Cohort | | P-Value | Std diff. |
|  |  | 1 2 | F41.9 | Anxiety disorder, unspecified |  | | 3,669 2,057 | 17.0% 10.1% | | <0.001 | 0.205 |
|  |  | 1 2 | F41.1 | Generalized anxiety disorder |  | | 1,643 943 | 7.6% 4.6% | | <0.001 | 0.126 |
|  |  | 1 2 | F41.8 | Other specified anxiety disorders |  | | 667 293 | 3.1% 1.4% | | <0.001 | 0.112 |
|  |  | 1 2 | F33.1 | Major depressive disorder, recurrent, moderate |  | | 751 422 | 3.5% 2.1% | | <0.001 | 0.087 |
|  |  | 1 2 | F33.2 | Major depressive disorder, recurrent severe without psychotic features |  | | 564 358 | 2.6% 1.8% | | <0.001 | 0.059 |
|  |  | 1 2 | F33.9 | Major depressive disorder, recurrent, unspecified |  | | 350 221 | 1.6% 1.1% | | <0.001 | 0.047 |
|  |  | 1 2 | F10.1 | Alcohol abuse |  | | 341 294 | 1.6% 1.4% | | 0.225 | 0.012 |
|  |  | 1 2 | F10.2 | Alcohol dependence |  | | 169 195 | 0.8% 1.0% | | 0.061 | 0.018 |
|  |  | 1 2 | F17 | Nicotine dependence |  | | 1,381 889 | 6.4% 4.4% | | <0.001 | 0.091 |
|  |  | 1 2 | G47 | Sleep disorders |  | | 1,516 1,114 | 7.0% 5.5% | | <0.001 | 0.066 |
|  | Laboratory | | | | | | | | | | |
|  |  | Cohort | |  | Mean ± SD | | Patients | % of Cohort | | P-Value | Std diff. |
|  |  | 1 2 | 9083 | BMI | 26.1 +/- 6.8 25.0 +/- 7.4 | | 6,842 3,636 | 31.8% 17.8% | | <0.001 | 0.153 |
|  |  | 1 2 |  | 0 - 0 kg/m2 |  | | 6,844 3,636 | 31.8% 17.8% | | <0.001 | 0.328 |
|  | Visits | | | | | | | | | | |
|  |  | Cohort | |  | Mean ± SD | | Patients | % of Cohort | | P-Value | Std diff. |
|  |  | 1 2 | 9083 | Visit Ambulatory |  | | 12,930 9,012 | 74.5% 51.9% | | <0.001 | 0.482 |
|  |  |  |  |  |  | |  |  | |  |  |
| Cohort 1 (N = 17,352) and cohort 2 (N = 17,352) characteristics after propensity score matching | | | | | | | | | | | |
|  | Demographics | | | | | | | | | | |
|  |  | Cohort | |  | Mean ± SD | | Patients | % of Cohort | | P-Value | Std diff. |
|  |  | 1 2 | Age | Current Age | 30.9 +/- 12.6 30.8 +/- 15.0 | | 17,352 17,352 | 100% 100% | | 0.587 | 0.006 |
|  |  | 1 2 | AI | Age at Index | 27.1 +/- 11.9 27.0 +/- 14.3 | | 17,352 17,352 | 100% 100% | | 0.865 | 0.002 |
|  |  | 1 2 | 2106-3 | White |  | | 12,236 12,536 | 70.5% 72.2% | | <0.001 | 0.038 |
|  |  | 1 2 | UNK | Unknown Race |  | | 2,150 1,994 | 12.4% 11.5% | | 0.010 | 0.028 |
|  |  | 1 2 | UN | Unknown Ethnicity |  | | 3,083 2,857 | 17.8% 16.5% | | 0.001 | 0.035 |
|  |  | 1 2 | 2186-5 | Not Hispanic or Latino |  | | 12,790 13,011 | 73.7% 75.0% | | 0.007 | 0.029 |
|  |  | 1 2 | 2135-2 | Hispanic or Latino |  | | 1,479 1,484 | 8.5% 8.6% | | 0.923 | 0.001 |
|  |  | 1 2 | 2054-5 | Black or African American |  | | 1,544 1,454 | 8.9% 8.4% | | 0.085 | 0.018 |
|  |  | 1 2 | 2028-9 | Asian |  | | 360 347 | 2.1% 2.0% | | 0.621 | 0.005 |
|  | Diagnosis | | | | | | | | | | |
|  |  | Cohort | |  | Mean ± SD | | Patients | % of Cohort | | P-Value | Std diff. |
|  |  | 1 2 | F41.9 | Anxiety disorder, unspecified |  | | 2,103 2,040 | 12.1% 11.8% | | 0.297 | 0.011 |
|  |  | 1 2 | F41.1 | Generalized anxiety disorder |  | | 1,001 930 | 5.8% 5.4% | | 0.096 | 0.018 |
|  |  | 1 2 | F41.8 | Other specified anxiety disorders |  | | 334 293 | 1.9% 1.7% | | 0.098 | 0.018 |
|  |  | 1 2 | F33.1 | Major depressive disorder, recurrent, moderate |  | | 431 417 | 2.5% 2.4% | | 0.626 | 0.005 |
|  |  | 1 2 | F33.2 | Major depressive disorder, recurrent severe without psychotic features |  | | 373 346 | 2.1% 2.0% | | 0.309 | 0.011 |
|  |  | 1 2 | F33.9 | Major depressive disorder, recurrent, unspecified |  | | 223 213 | 1.3% 1.2% | | 0.630 | 0.005 |
|  |  | 1 2 | F10.1 | Alcohol abuse |  | | 249 255 | 1.4% 1.5% | | 0.788 | 0.003 |
|  |  | 1 2 | F10.2 | Alcohol dependence |  | | 139 145 | 0.8% 0.8% | | 0.721 | 0.004 |
|  |  | 1 2 | F17 | Nicotine dependence |  | | 885 850 | 5.1% 4.9% | | 0.389 | 0.009 |
|  |  | 1 2 | G47 | Sleep disorders |  | | 1,073 1,028 | 6.2% 5.9% | | 0.311 | 0.011 |
|  | Laboratory | | | | | | | | | | |
|  |  | Cohort | |  | Mean ± SD | | Patients | % of Cohort | | P-Value | Std diff. |
|  |  | 1 2 | 9083 | BMI | 26.0 +/- 6.9 25.0 +/- 7.4 | | 3,724 3,633 | 21.5% 20.9% | | <0.001 | 0.138 |
|  |  | 1 2 |  | 0 - 0 kg/m2 |  | | 3,726 3,633 | 21.5% 20.9% | | 0.222 | 0.013 |
|  | Visits | | | | | | | | | | |
|  |  | Cohort | |  | Mean ± SD | | Patients | % of Cohort | | P-Value | Std diff. |
|  |  | 1 2 | 9083 | Visit Ambulatory |  | | 11,349 11,452 | 65.4% 66.0% | | 0.291 | 0.012 |

Trans Men On Testosterone Therapy vs Trans Men Naïve to Hormone Therapy

| Cohort 1 and cohort 2 patient count before and after propensity score matching | | | | | | | | | | | |
| --- | --- | --- | --- | --- | --- | --- | --- | --- | --- | --- | --- |
|  | | Cohort | | | Patient count before matching | | | | Patient count after matching | | |
|  | | 1 - Trans Men On Testosterone Therapy | | | 23,018 | | | | 22,037 | | |
|  | | 2 - Trans Men Naïve to Hormone Therapy | | | 34,125 | | | | 22,037 | | |
| Propensity score density function - Before and after matching (cohort 1 - black, cohort 2 - grey) | | | | | | | | | | | |
|  |  | 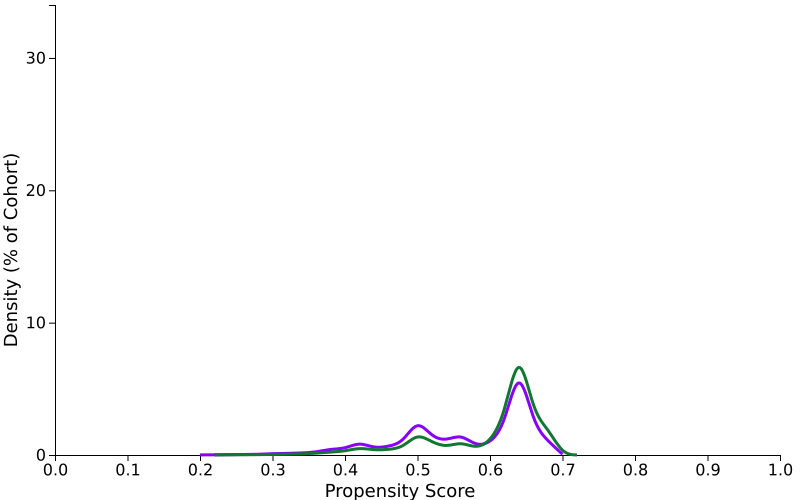 | | | | 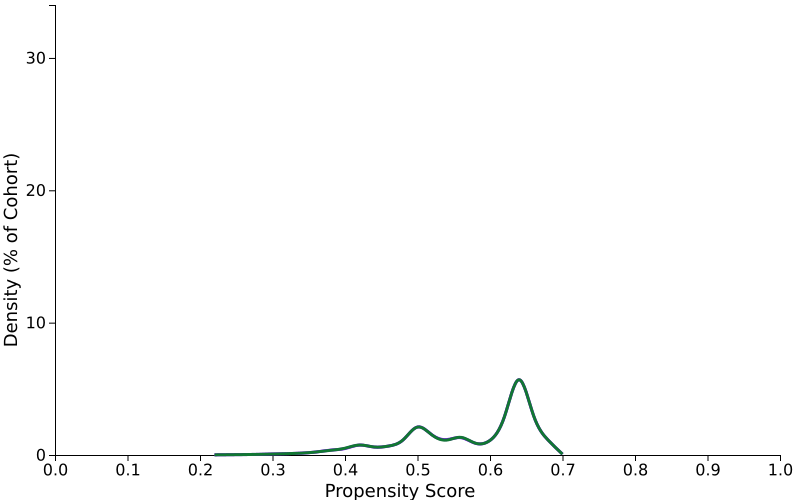 | | | | | |
| Cohort 1 (N = 23,018) and cohort 2 (N = 34,125) characteristics before propensity score matching | | | | | | | | | | | |
|  | Demographics | | | | | | | | | | |
|  |  | Cohort | |  | Mean ± SD | | Patients | % of Cohort | | P-Value | Std diff. |
|  |  | 1 2 | Age | Current Age | 26.9 +/- 10.8 26.8 +/- 13.6 | | 22,989 33,907 | 100% 100% | | 0.388 | 0.008 |
|  |  | 1 2 | AI | Age at Index | 23.4 +/- 10.1 23.2 +/- 12.7 | | 22,989 33,907 | 100% 100% | | 0.045 | 0.017 |
|  |  | 1 2 | 2106-3 | White |  | | 16,615 23,667 | 72.3% 69.8% | | <0.001 | 0.055 |
|  |  | 1 2 | UNK | Unknown Race |  | | 2,845 4,882 | 12.4% 14.4% | | <0.001 | 0.059 |
|  |  | 1 2 | UN | Unknown Ethnicity |  | | 3,702 6,494 | 16.1% 19.2% | | <0.001 | 0.080 |
|  |  | 1 2 | 2186-5 | Not Hispanic or Latino |  | | 17,301 24,601 | 75.3% 72.6% | | <0.001 | 0.062 |
|  |  | 1 2 | 2135-2 | Hispanic or Latino |  | | 1,986 2,812 | 8.6% 8.3% | | 0.145 | 0.012 |
|  |  | 1 2 | 2054-5 | Black or African American |  | | 1,543 2,373 | 6.7% 7.0% | | 0.185 | 0.011 |
|  |  | 1 2 | 2028-9 | Asian |  | | 547 796 | 2.4% 2.3% | | 0.806 | 0.002 |
|  | Diagnosis | | | | | | | | | | |
|  |  | Cohort | |  | Mean ± SD | | Patients | % of Cohort | | P-Value | Std diff. |
|  |  | 1 2 | F41.9 | Anxiety disorder, unspecified |  | | 5,068 4,915 | 22.0% 14.5% | | <0.001 | 0.196 |
|  |  | 1 2 | F41.1 | Generalized anxiety disorder |  | | 2,471 2,544 | 10.7% 7.5% | | <0.001 | 0.113 |
|  |  | 1 2 | F41.8 | Other specified anxiety disorders |  | | 930 861 | 4.0% 2.5% | | <0.001 | 0.084 |
|  |  | 1 2 | F33.1 | Major depressive disorder, recurrent, moderate |  | | 1,213 1,060 | 5.3% 3.1% | | <0.001 | 0.107 |
|  |  | 1 2 | F33.2 | Major depressive disorder, recurrent severe without psychotic features |  | | 910 1,008 | 4.0% 3.0% | | <0.001 | 0.054 |
|  |  | 1 2 | F33.9 | Major depressive disorder, recurrent, unspecified |  | | 522 547 | 2.3% 1.6% | | <0.001 | 0.048 |
|  |  | 1 2 | F10.1 | Alcohol abuse |  | | 215 217 | 0.9% 0.6% | | <0.001 | 0.033 |
|  |  | 1 2 | F10.2 | Alcohol dependence |  | | 126 131 | 0.5% 0.4% | | 0.005 | 0.024 |
|  |  | 1 2 | F17 | Nicotine dependence |  | | 986 742 | 4.3% 2.2% | | <0.001 | 0.119 |
|  |  | 1 2 | G47 | Sleep disorders |  | | 1,762 2,060 | 7.7% 6.1% | | <0.001 | 0.063 |
|  | Laboratory | | | | | | | | | | |
|  |  | Cohort | |  | Mean ± SD | | Patients | % of Cohort | | P-Value | Std diff. |
|  |  | 1 2 | 9083 | BMI | 27.1 +/- 7.6 25.4 +/- 7.4 | | 7,558 6,918 | 32.9% 20.4% | | <0.001 | 0.231 |
|  |  | 1 2 |  | 0 - 0 kg/m2 |  | | 7,560 6,924 | 32.9% 20.4% | | <0.001 | 0.285 |
|  | Visits | | | | | | | | | | |
|  |  | Cohort | |  | Mean ± SD | | Patients | % of Cohort | | P-Value | Std diff. |
|  |  | 1 2 | 9083 | Visit Ambulatory |  | | 16,858 12,010 | 76.5% 54.5% | | <0.001 | 0.477 |
| Cohort 1 (N = 22,037) and cohort 2 (N = 22,037) characteristics after propensity score matching | | | | | | | | | | | |
|  | Demographics | | | | | | | | | | |
|  |  | Cohort | |  | Mean ± SD | | Patients | % of Cohort | | P-Value | Std diff. |
|  |  | 1 2 | Age | Current Age | 26.9 +/- 10.8 26.8 +/- 13.2 | | 22,037 22,037 | 100% 100% | | 0.321 | 0.009 |
|  |  | 1 2 | AI | Age at Index | 23.4 +/- 10.2 23.3 +/- 12.4 | | 22,037 22,037 | 100% 100% | | 0.501 | 0.006 |
|  |  | 1 2 | 2106-3 | White |  | | 15,894 16,108 | 72.1% 73.1% | | 0.022 | 0.022 |
|  |  | 1 2 | UNK | Unknown Race |  | | 2,730 2,543 | 12.4% 11.5% | | 0.006 | 0.026 |
|  |  | 1 2 | UN | Unknown Ethnicity |  | | 3,568 3,413 | 16.2% 15.5% | | 0.043 | 0.019 |
|  |  | 1 2 | 2186-5 | Not Hispanic or Latino |  | | 16,585 16,708 | 75.3% 75.8% | | 0.173 | 0.013 |
|  |  | 1 2 | 2135-2 | Hispanic or Latino |  | | 1,884 1,916 | 8.5% 8.7% | | 0.587 | 0.005 |
|  |  | 1 2 | 2054-5 | Black or African American |  | | 1,500 1,522 | 6.8% 6.9% | | 0.678 | 0.004 |
|  |  | 1 2 | 2028-9 | Asian |  | | 527 531 | 2.4% 2.4% | | 0.901 | 0.001 |
|  | Diagnosis | | | | | | | | | | |
|  |  | Cohort | |  | Mean ± SD | | Patients | % of Cohort | | P-Value | Std diff. |
|  |  | 1 2 | F41.9 | Anxiety disorder, unspecified |  | | 4,599 4,683 | 20.9% 21.3% | | 0.326 | 0.009 |
|  |  | 1 2 | F41.1 | Generalized anxiety disorder |  | | 2,261 2,228 | 10.3% 10.1% | | 0.603 | 0.005 |
|  |  | 1 2 | F41.8 | Other specified anxiety disorders |  | | 840 787 | 3.8% 3.6% | | 0.181 | 0.013 |
|  |  | 1 2 | F33.1 | Major depressive disorder, recurrent, moderate |  | | 1,076 1,020 | 4.9% 4.6% | | 0.210 | 0.012 |
|  |  | 1 2 | F33.2 | Major depressive disorder, recurrent severe without psychotic features |  | | 847 842 | 3.8% 3.8% | | 0.901 | 0.001 |
|  |  | 1 2 | F33.9 | Major depressive disorder, recurrent, unspecified |  | | 490 472 | 2.2% 2.1% | | 0.557 | 0.006 |
|  |  | 1 2 | F10.1 | Alcohol abuse |  | | 199 172 | 0.9% 0.8% | | 0.159 | 0.013 |
|  |  | 1 2 | F10.2 | Alcohol dependence |  | | 117 108 | 0.5% 0.5% | | 0.547 | 0.006 |
|  |  | 1 2 | F17 | Nicotine dependence |  | | 852 734 | 3.9% 3.3% | | 0.003 | 0.029 |
|  |  | 1 2 | G47 | Sleep disorders |  | | 1,647 1,591 | 7.5% 7.2% | | 0.307 | 0.010 |
|  | Laboratory | | | | | | | | | | |
|  |  | Cohort | |  | Mean ± SD | | Patients | % of Cohort | | P-Value | Std diff. |
|  |  | 1 2 | 9083 | BMI | 27.1 +/- 7.6 25.4 +/- 7.3 | | 6,779 6,786 | 30.8% 30.8% | | <0.001 | 0.228 |
|  |  | 1 2 |  | 0 - 0 kg/m2 |  | | 6,781 6,792 | 30.8% 30.8% | | 0.910 | 0.001 |
|  | Visits | | | | | | | | | | |
|  |  | Cohort | |  | Mean ± SD | | Patients | % of Cohort | | P-Value | Std diff. |
|  |  | 1 2 | 9083 | Visit Ambulatory |  | | 16,572 16,601 | 75.2% 75.3% | | 0.291 | 0.012 |

Footnotes: SD=standard deviation, Std Diff=standardized mean difference, BMI=body mass index. Diagnosis codes for PSM are reflective of risk factors for chronic pain development as described in the following reference.

Mills SEE, Nicolson KP, Smith BH. Chronic pain: a review of its epidemiology and associated factors in population-based studies. Br J Anaesth 2019;123(2):e273–e283; doi: 10.1016/j.bja.2019.03.023.
